# Prenatal diagnosis of fetal neurogenic megacystis associated with lethal congenital contractural syndrome 2

**DOI:** 10.1515/crpm-2024-0024

**Published:** 2024-10-09

**Authors:** Christos G. Hatjis, Wendy J. Sturtz, Jillian Taylor, Elizabeth Melchionna, Kerry K. Brown

**Affiliations:** Bayhealth Maternal Fetal Medicine Associates, Department of OBGYN, 21665 Bayhealth Medical Center, Dover, DE, USA; Neonatal Transport Team, Infant Maternal Pediatric Advanced Care Team (IMPACT), Christiana Care Health System, Wilmington, DE, USA; Nemours Children’s Hospital, Wilmington, DE, USA; HNL Genomics/Connective Tissue Gene Tests, Allentown, PA, USA

**Keywords:** prenatal diagnosis of LCCS2, arhrogryposis multiplex, neurogenic megacystis, nonobstructive fetal megacystis

## Abstract

**Objectives:**

To describe the prenatal diagnosis, unique clinical features, clinical and genetic evaluation, and the pregnancy and neonatal course of two siblings affected by Lethal Congenital Contractural Syndrome 2 (LCCS2).

**Case presentation:**

We present two cases of LCCS2, a rare autosomal recessive disorder in the arthrogryposis multiplex spectrum of syndromes whose *sine qua non* feature is the presence of nonobstructive, neurogenic megacystis. The prenatal diagnosis of this syndrome has not been previously reported. This syndrome has been previously studied in detail in an Israeli-Bedouin kindred but it has not been reported in the Americas.

**Conclusions:**

These two cases illustrate the diagnostic and therapeutic dilemmas associated with this rare genetic abnormality. LCCS2 can be seen in other patient populations besides Israeli-Bedouin. They also suggest the presence of phenotypic variability in the clinical outcomes. Finally, they underscore the need for specialized diagnostic capabilities, the involvement of multidisciplinary teams to support challenging family situations, and the need for shared decision-making.

## Introduction

We report two siblings affected by Lethal Congenital Contracture or Contractural Syndrome 2 (LCCS2), a rare autosomal recessive disorder in the arthrogryposis multiplex spectrum of syndromes [1] due to a specific mutation. LCCS2 subtype is most often associated with neonatal death. Phenotypically, affected persons show multiple joint contractures thought to be due to motor neuron atrophy or degeneration in the anterior horn of the spinal cord. Its *sine qua non* feature is the presence of nonobstructive megacystis-neurogenic bladder [1, 2]. Prenatal diagnosis of LCCS2 has not been previously reported. This rare syndrome has been studied in detail in an Israeli-Bedouin kindred [2] but has not been detected or reported in the Americas. We describe the clinical features, diagnostic and genetic evaluation, and the pregnancy and neonatal course of these siblings.

## Case presentation

During her current pregnancy, this 35-year-old gravida 8 para 4-0-3-3 patient was referred to Maternal Fetal Medicine for evaluation of an abnormal ultrasound examination at 19 weeks and 6 days gestation. Patient reported that all her pregnancies were the results of a nonconsanguineous, monogamous relationship. Her three first-trimester pregnancy losses could have been associated with antiphospholipid syndrome. She had three older living children who were healthy. Parental genetic and family histories were noncontributory except as follows.

Three years earlier, we had evaluated this patient at 33 weeks gestation because of concerns about fetal growth restriction and possible skeletal abnormalities. Patient’s earlier ultrasound examinations had been done elsewhere. The ultrasound examination that was done in the mid second trimester reported no significant fetal structural abnormalities. At the time of our initial ultrasound examination at 33 weeks gestation, we noted polyhydramnios, fetal growth restriction, suspected skeletal dysplasia with shortened long bones, syndactyly, and dilated loops of bowel. The following week, we also noted the development of fetal megacystis. The patient delivered in a level III/IV perinatal center. That female baby died at 6 weeks of age following a complex neonatal course. An autopsy was not performed. The patient was aware that the baby had some type of genetic abnormality and skeletal problems but provided no detailed information regarding the specific diagnosis or the possibility of recurrence in the current pregnancy.

According to her medical records, the first affected sibling’s delivery was at 37 weeks. Apgars were 1 at 1 min, 3 at 5 min, and 6 at 10 min. Growth parameters included weight 1,820 g (2 %), head circumference 33 cm (49 %), and length 46 cm (24 %). The baby had flexion contractures and required respiratory resuscitation including intubation and ventilator support. They described limited range of motion of knees and ankles. Cranial ultrasound and echocardiogram were structurally normal. Renal ultrasound revealed an overly distended bladder, bilateral distal ureteral dilatation, and left renal calyceal dilation consistent with urinary tract dilation classification P3. Baby was transferred after initial stabilization and evaluation to the neonatal intensive care unit in a local children’s hospital for subspecialty support and ongoing care. The initial SNP (single-nucleotide polymorphism) microarray genetic workup at that time was negative except for regions of homozygosity (ROH) involving several chromosomes. Targeted genetic evaluation for connective tissue disorders was carried out at CTGT (Connective Tissue Gene Testing/HNL Genomics). The proband was tested with CTGT’s congenital contracture syndrome extended panel, which analyzes the coding exons and exon boundaries of the following genes through Next Generation Sequencing (NGS) and copy number variant (CNV) analysis: *ADCY6*, *ADGRG6*, *CHRNA1*, *CHRND*, *CHRNG*, *CNTNAP1*, *DNM2*, *DOK7*, *ECEL1*, *ERBB3*, *FBN2*, *GLDN*, *GLE1*, *LGI4*, *LMNA*, *MUSK*, *MYBPC1*, *MYH3*, *MYH8*, *NALCN*, *NEK9*, *PIEZO2*, *PIP5K1C*, *RAPSN*, *TNNI2*, *TNNT3*, *TPM2*, *VIPAS39*, *VPS33B*, *ZBTB42*, *ZMPSTE24*. A homozygous pathogenic *ERBB3* IVS10 c.1184-9A>G (transcript NM_001982) variant was detected in this patient, and was confirmed by Sanger sequencing.

**Figure 1:**
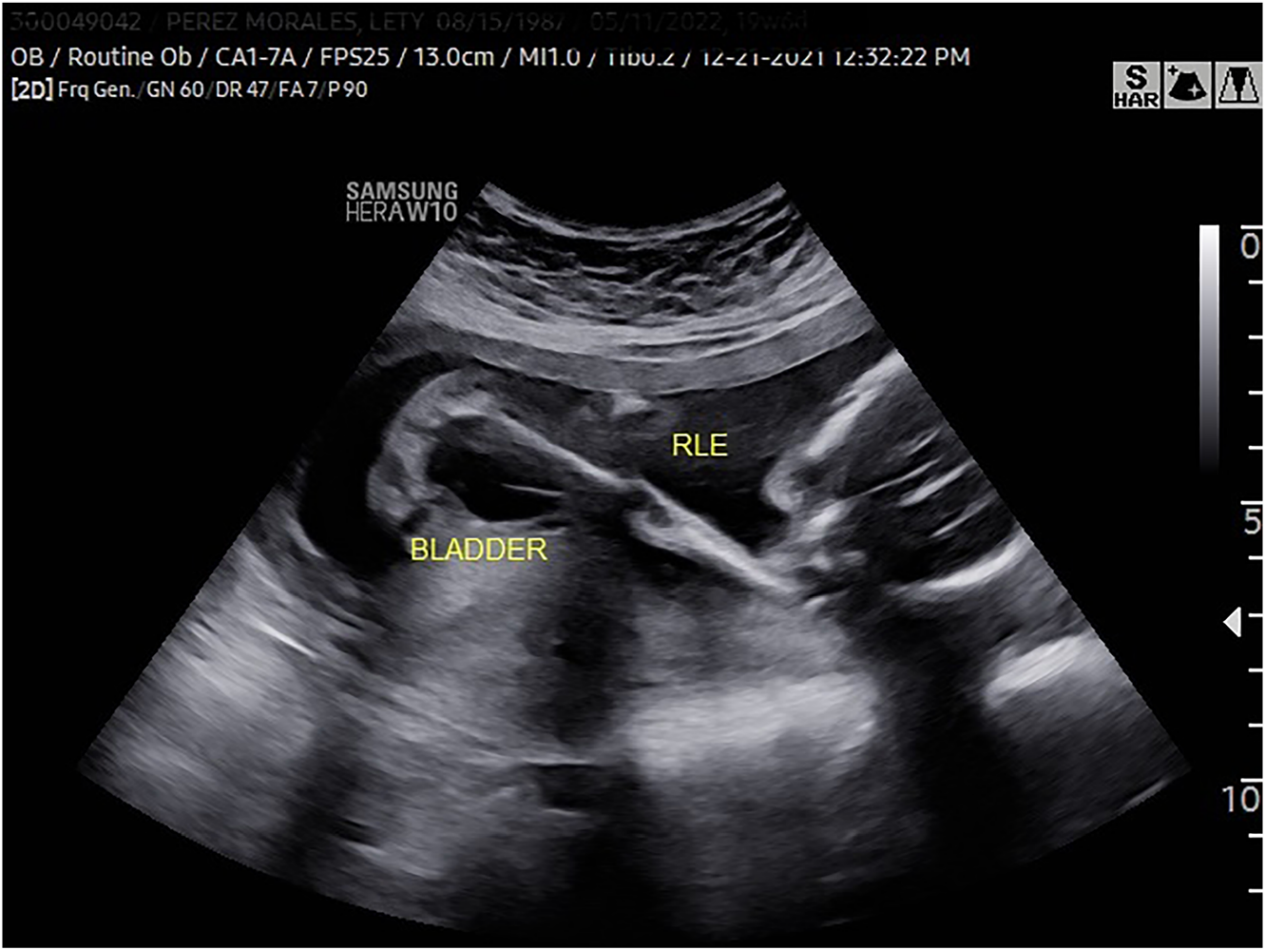
Abnormal positioning and fixed joints (knee and hip) of the fetal right lower extremity.

**Figure 2:**
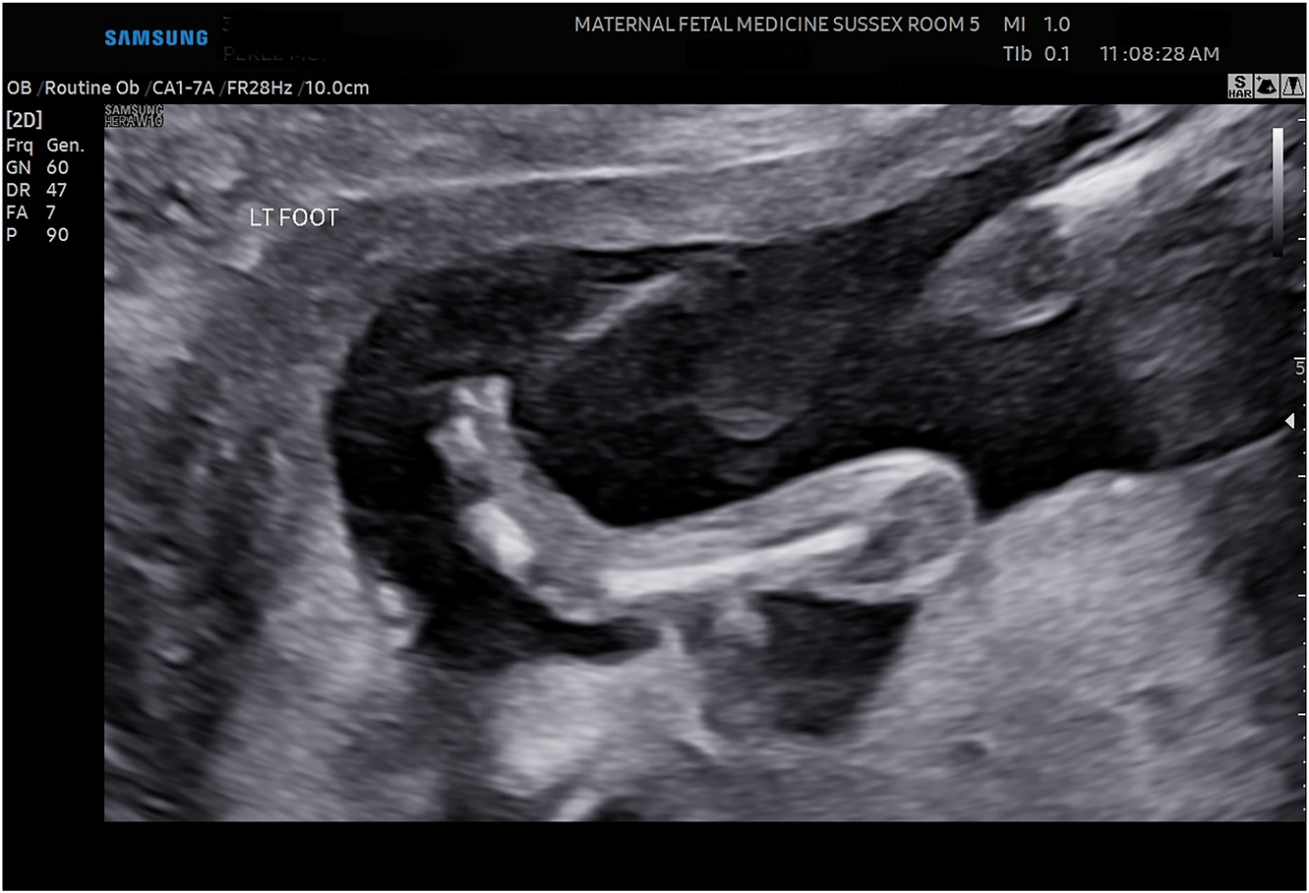
Fetal left clubfoot.

**Figure 3:**
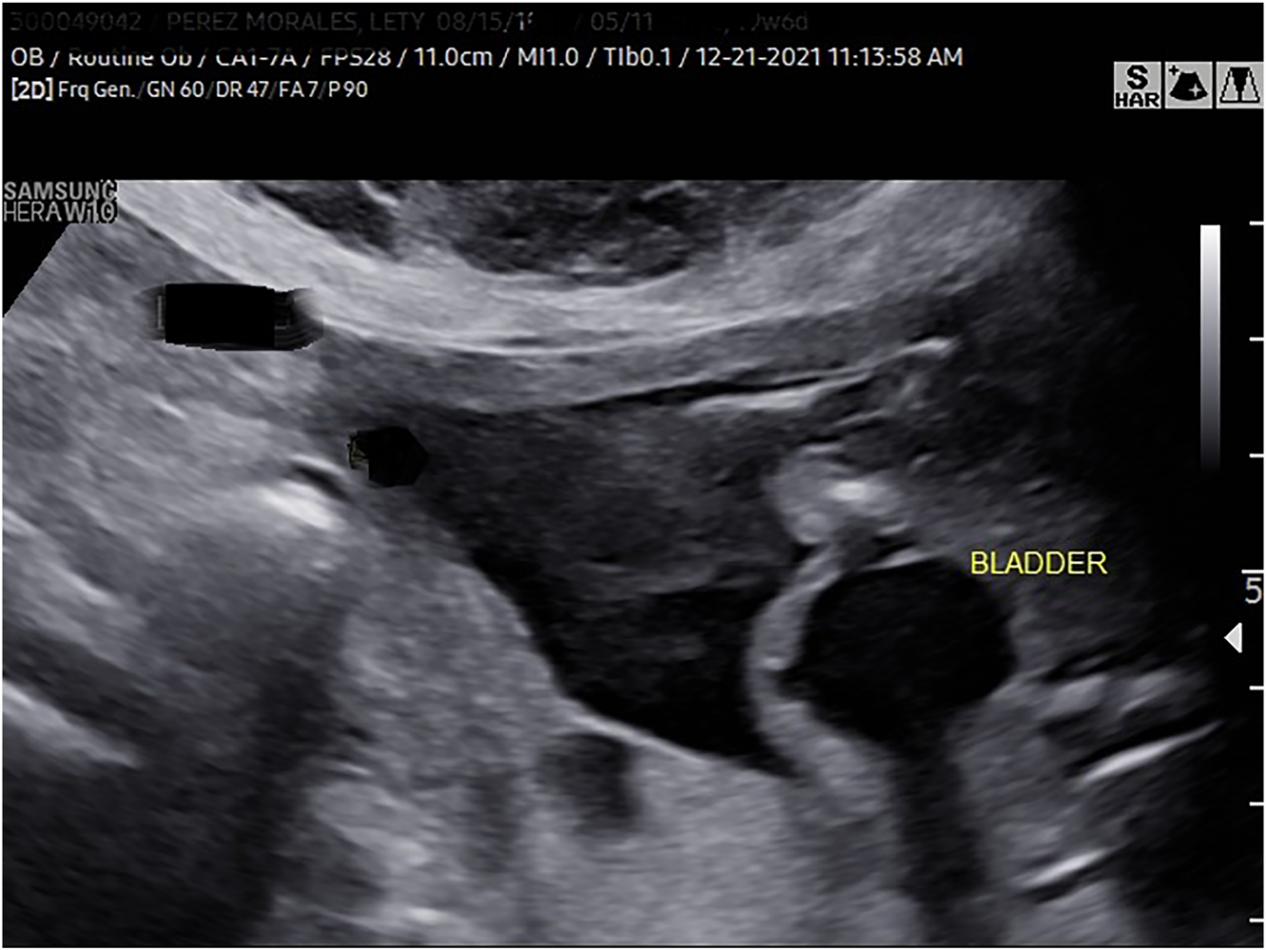
Fetal megacystis with keyhole appearance.

In the current pregnancy at 19 weeks and 6 days gestation, we detected fetal growth restriction, as well as an abnormal skeletal system survey consisting of fixed joints, abnormal positioning of the lower extremities (Figure 1), limited mobility and clubfoot (Figure 2), among others. In addition, we noted megacystis (Figure 3) with a keyhole appearance but normal appearing kidneys and normal amniotic fluid volume. We considered arthrogryposis as the primary diagnosis and possible partial urethral obstruction. Patient underwent a diagnostic amniocentesis to evaluate the fetal chromosomal karyotype, perform microarray analysis and additional testing, if indicated.

Routine chromosomal karyotype and microarray analysis were consistent with a normal female fetus. However, microarray showed a high density of short (1–8 Mb) regions of allele homozygosity (ROH) throughout the genome. This was consistent with a limited gene pool as seen in isolated populations. Based on that information and the ultrasonographic clinical picture, we requested CTGT lab to perform targeted analysis on the current case. Targeted testing for this particular *ERBB3* variant was performed on the amniocytes via Sanger sequencing. This fetus carried the same homozygous pathogenic ERBB3 variant as her deceased sister, consistent with LCCS2. The *ERBB3* gene is present in the following ROH: chr12:51353850-60083096.

We followed this fetus with serial ultrasound examinations showing progressive fetal growth restriction, persistent megacystis and features consistent with arthrogryposis. She had consultations with pediatric urology and a Perinatal Palliative Care Team including representatives from neonatology, pediatrics, Chaplaincy, among others. Given the family’s previous experience and the counseled expectations for infants born with LCCS2, patient requested a supported comfort care model of care after birth. This female infant was delivered in the same delivery hospital as her deceased sister at 37 weeks, with growth parameters of birth weight 1,830 g (0.5 %), length 42.5 cm (21 %), and head circumference 33.5 cm (36 %). Apgars were 1 at 1 min, 3 at 5 min, 6 at 10 min, and 8 at 15 min. Despite expectations to allow a natural death after birth, she continued to have respiratory drive and live. The family’s goals of care pivoted to accept neonatal intensive level care with perceived non-aggressive interventions such as nasal cannula oxygen support, enteral feedings via nasogastric tube, thermoregulation support, and Foley catheter. Renal ultrasound showed normal kidneys with a distended bladder with mild wall thickening. She required an indwelling Foley catheter for adequate bladder emptying. After a prolonged course, including ongoing noninvasive respiratory support and transfer to the local children’s hospital for ongoing care, the second affected sibling also died on the 96th day of life from respiratory failure and presumed pneumonia. An autopsy was not done.

## Discussion

We report the first prenatal diagnosis of LCCS2 in a family from the Americas. LCCS2 is a rare, severe and lethal arthrogryposis multiplex syndrome. Lethal congenital contracture syndromes (LCCS) are autosomal recessive. In contrast, distal arthrogryposis syndromes are almost all autosomal dominant [1]. 11 subtypes of the LCCS have been reported [3]. Major characteristics include akinesia of the limbs, contractures, pterygium formation, muscle atrophy, hydrops, among others. In LCCS1 and LCCS2, degeneration of motor neuron cells and/or atrophy of the anterior horn neurons of the spinal column are observed [1, 2].

LCCS2 is unique from other subtypes due to the presence of a distended urinary bladder and other urinary abnormalities [2]. In addition, craniofacial and ocular deformities have been reported. Hydrops and pterygia have not been reported in this subtype. The duration of pregnancy appears to be normal. Neonatal survival lasts few weeks or rarely a few months.

LCCS1 has been reported and well characterized in Finnish families (OMIM #253310), [5]. This disorder is caused by mutations in the *GLE1* gene, which has been reported in homozygous and compound heterozygous states. GLE1 is an essential mRNA export factor but also plays a role in initiation and termination of protein translation. Phenotypically, it is associated with arthrogryposis involving proximal and distal joints, hydrops, among others. Anterior horn motor cell apoptosis is noted. However, depending on the particular mutation present, not all cases result in neonatal death; several long-term survivors have been reported. Some have advocated renaming this syndrome as Arthrogryposis with Anterior Horn Cell Disease, since not all cases are lethal. These findings are consistent with phenotypic variability and suggest a specific genotype/phenotype correlation [6]. Notably, LCCS1 is not associated with megacystis.

In the obstetrical literature, megacystis with urethral dilation (keyhole appearance) is usually associated with obstructive uropathy. Megacystis without dilated urethra has been reported in megacystis microcolon intestinal hypoperistalsis syndrome. Megacystis has not been considered part of an arthrogryposis syndrome. However, in LCCS2, megacystis is a unique feature and represents a diagnostic clue. In LCCS2, megacystis is not due to obstruction, despite its sonographic appearance, but rather to neurogenic causes. Given the absence of megacystis in LCCS1, it is not likely that damage of the ventral horn motor neurons of the sacral spinal cord that innervate the external striated urethral sphincter is responsible for the neurogenic bladder/megacystis. Dysregulation of the *ERRB3* pathway has been associated with GI dysmotility and can manifest as Hirschprung disease, hypoganglionosis, among others, thus offering an alternative plausible explanation [7, 8]. Other mechanisms may be involved.

LCCS2 was initially reported in two Israeli-Bedouin kindreds [2, 4]. Affected persons are homozygous for the ERBB3 IVS10 c.1184-9A>G mutation. The cases we describe herein are the first who have been reported in the Americas. Although we do not have parental genetic testing to confirm that the parents are carriers for the *ERRB3* mutation, the family/reproductive history and the fetal microarray results lead us to conclude that the parents of the two affected fetuses/neonates are obligate heterozygous carriers and that some degree of relatedness/consanguinity is present.

ERRB3 (Her3) encodes an epidermal growth factor receptor. It is an activator of the phosphatidylinositol-3-kinase/Akt pathway that regulates cell survival, proliferation, adhesion, differentiation and endocytosis of synaptic vesicle proteins. It is essential for the generation of precursors of Schwann cells that normally accompany peripheral axons of motor neurons. Although gain of function mutations in members of the epidermal growth factor tyrosine kinase receptor family have been associated with predilection to cancer, this particular mutation of LCCS2 results in loss of function and the associated human phenotype [2].

A functional study performed by Narkis et al. [2] determined that the pathogenic *ERBB3* c.1184-9A>G variant (reported as IVS10-8A-G) does not alter the original acceptor site, but generates an alternative acceptor site, resulting in an insertion of 8 bases between exons 10 and 11, which was detected in the fibroblasts of affected patients. This aberrant splicing is predicted to cause a frameshift and lead to nonsense mediated decay with decreased or absent protein product in family members affected with LCCS2. The hypothesis is that “homozygous ERBB3 mutant embryos lack Schwann-cell precursors and Schwann cells that normally accompany peripheral axons of sensory and motor neurons” [2]. Neuronal apoptosis has been demonstrated in the LCCS2 phenotype [4].

The first affected member of this family presented with diagnostic dilemmas. The initial genetic workup was noninformative. Eventually, the correct diagnosis was established with the help of a specialty lab. This testing is not widely available. The diagnostic work up of the second affected family member benefited from the information we eventually obtained about her deceased sister and allowed for targeted testing of the *ERBB3* mutation. Notably, it appears that these two LCCS2 sibling cases showed some phenotypic variability.

Pregnancy management is difficult for providers and families facing a fetal LCCS2 diagnosis with respect to counseling about pregnancy continuation vs. termination as well as preparing for the peripartum management of the neonate given the limited literature regarding clinical outcomes and life expectancy in patients with LCCS2. Perinatal palliative care is principled to support families through this uncertainty using shared decision-making to establish goals of care.

Patient education is also critical in these situations given the implications for the entire family. We counseled the parents about the recurrence risk of 25 %. We also recommended that they inform other family members about the possibility that they may be carriers for this mutation. Finally, parents understood that each one of their living children could be carriers. Given the rarity of this mutation and the question of relatedness, we recommended that all family members pursue genetic testing.

In summary, these two cases illustrate the diagnostic and therapeutic dilemmas associated with this rare genetic abnormality. In addition, they show that LCCS2 can be seen in other patient populations besides the ones already reported in the literature. They also suggest the presence of phenotypic variability in the clinical outcomes. Finally, they underscore the need for specialized diagnostic capabilities, the involvement of multidisciplinary teams to support challenging family situations, and the need for shared decision-making.
